# The multidimensional roles and mechanisms of exercise intervention in pediatric oncology

**DOI:** 10.3389/fimmu.2026.1784767

**Published:** 2026-03-13

**Authors:** Shengting Dai, Zhenyu Yang, Yang Wu, Maoqi Wu, Murui Ma, Xiaonan Zhang, Haoyu Tang, Xinming Ye, Mei Du

**Affiliations:** 1 School of Sports Science and Engineering, East China University of Science and Technology, Shanghai, China; 2 Ningde Normal University, Ningde, Fujian, China

**Keywords:** exercise intervention, immune modulation, pediatric cancer, personalized therapy, physiological and psychological recovery, tumor microenvironment

## Abstract

The importance of exercise interventions in pediatric oncology is steadily increasing. Their value lies not only in alleviating treatment-related adverse effects but also in supporting physiological recovery, psychological adjustment, and improvements in cognitive function. As research continues to reveal the effects of exercise on the tumor microenvironment, immune modulation, and energy metabolism, its role in pediatric cancer care is expanding from a rehabilitative adjunct to a comprehensive intervention with potential therapeutic relevance. Exercise can enhance tumor-related biological processes by improving blood flow and tissue oxygenation, increasing immune cell activity, mitigating immunosuppression, and modulating glucose utilization and fatty acid metabolism, thereby influencing the energy supply to tumor cells. At the clinical level, exercise strengthens skeletal muscle, improves cardiopulmonary function, increases physical reserves, and reduces long-term side effects such as fatigue and frailty. It also contributes to emotional stability, strengthens self-efficacy, and ameliorates cognitive impairments including attention and memory deficits. Moreover, exercise may exert synergistic effects with chemotherapy, radiotherapy, and immunotherapy by improving treatment tolerance and amplifying overall therapeutic benefit. Despite its potential, the implementation of exercise interventions remains challenged by limited resources, variability in adherence, and age-related differences. Future research should aim to develop personalized exercise prescription systems tailored to pediatric needs, supported by interdisciplinary teams and intelligent monitoring tools to enhance feasibility and scientific rigor. With continued advances in basic research and the accumulation of clinical evidence, exercise is expected to play an increasingly systematic and sustained role in pediatric cancer treatment, providing crucial support for improving rehabilitation quality and long-term health outcomes.

## Graphical Abstract

**Figure:**
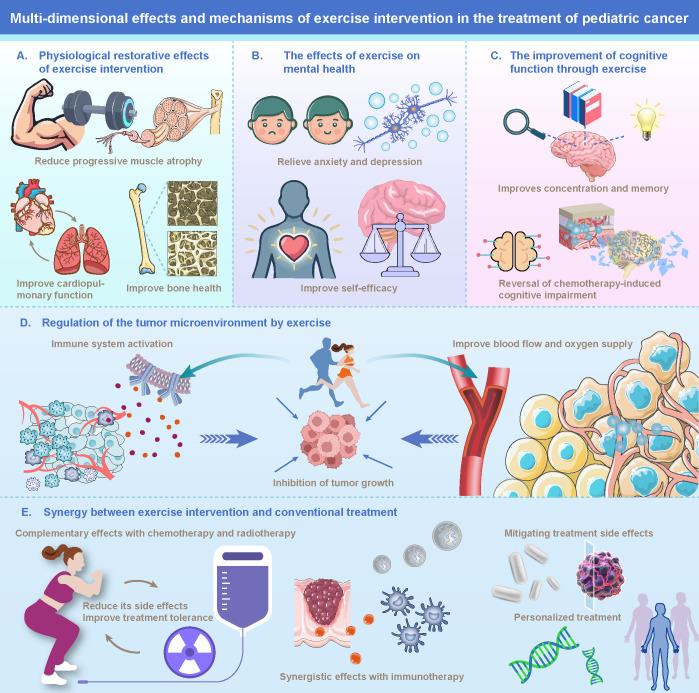
Exercise interventions in pediatric oncology are increasingly recognized as a comprehensive treatment strategy, offering multidimensional benefits that go beyond rehabilitation. These interventions contribute to physiological recovery by reducing muscle atrophy, enhancing cardiopulmonary health, and improving bone density. Additionally, exercise plays a crucial role in supporting mental health by alleviating anxiety, depression, and boosting self-efficacy. It also aids in reversing chemotherapy-induced cognitive impairments, improving concentration and memory. Furthermore, exercise positively influences the tumor microenvironment by enhancing blood flow and oxygen supply, inhibiting tumor growth, and boosting immune system activity. Finally, exercise demonstrates synergistic effects with conventional cancer treatments, such as chemotherapy, radiotherapy, and immunotherapy, by improving treatment tolerance, reducing side effects, and amplifying therapeutic efficacy. This multidimensional approach highlights exercise’s potential as a key strategy in enhancing both the physical and psychological recovery of pediatric cancer patients, optimizing overall treatment outcomes.

## 1 Introduction

### 1.1 The urgency of exercise in the treatment of pediatric oncology patients

Treating pediatric cancers remains a complex and pressing challenge in modern medicine. With ongoing advances in therapeutic technologies, survival rates for childhood cancer continue to improve. However, the side effects of treatment and the long-term sequelae still severely impact patients’ quality of life. Although conventional therapies such as chemotherapy, radiotherapy, and surgery offer substantial therapeutic benefits, they also impose significant physiological and psychological burdens. During treatment, pediatric patients often experience physical decline, muscle atrophy, impaired skeletal development, and reduced immune function (1). In the later stages of treatment, children frequently suffer from fatigue, depression, and anxiety, which seriously hinder the rehabilitation process and compromise overall well-being (2).

The side effects of current treatment modalities not only inflict significant physical harm on patients but also gradually exacerbate their psychological stress. This is particularly evident in pediatric patients, for whom the long-term fatigue, pain, and chronic symptoms following chemotherapy and radiotherapy make it difficult to return to a normal lifestyle and prevent them from engaging in everyday activities like their peers, thereby severely hindering both physical and psychological development (3). Against this backdrop, exercise intervention has garnered increasing attention within the medical community as an adjunctive therapy (4). Physical activity not only helps to alleviate treatment-related side effects but can also enhance the physical fitness of pediatric patients and support the recovery of physiological functions (5). Consequently, the integration of exercise interventions into pediatric oncology has emerged as a focal point in current medical research. Effectively mitigating the adverse impacts of cancer treatment through physical activity and improving patients’ quality of life have thus become pressing issues in need of resolution.

### 1.2 The therapeutic potential of exercise in pediatric oncology

Exercise interventions have demonstrated considerable potential in mitigating treatment-related side effects among pediatric cancer patients. Studies have shown that appropriate physical activity can significantly enhance physical strength, improve cardiopulmonary function, and effectively alleviate fatigue and muscle atrophy associated with cancer therapies (6). During treatment, prolonged bed rest or restricted mobility often leads to muscular wasting and reduced motor capacity in children, thereby impeding the recovery of physical function (7). Exercise interventions can strengthen muscular power and endurance, improve motor abilities, and support healthy skeletal development and growth, facilitating the restoration of pre-treatment physical fitness levels (8). In addition to physical benefits, exercise plays a critical role in promoting psychological well-being in pediatric oncology patients. Many children experience emotional disturbances such as anxiety and depression during treatment (9). Exercise, by stimulating the release of neurotransmitters like endorphins, can effectively reduce stress, alleviate anxiety, and enhance mood (10). Research indicates that moderate physical activity can improve psychological status and help pediatric patients cope more positively with the challenges of treatment (11). Moreover, exercise can strengthen immune function, improve overall cardiopulmonary performance, and reduce the risk of complications, thereby contributing to comprehensive health recovery (12). As a multidimensional therapeutic modality, exercise supports not only physical rehabilitation but also psychological resilience, making it an increasingly integral component of modern cancer care (13).

### 1.3 Modulation of the tumor microenvironment by exercise intervention and its role in personalized therapy

Exercise interventions can improve the overall condition of pediatric cancer patients at both physiological and psychological levels, while also offering new strategies for personalized therapy through modulation of the tumor microenvironment. The tumor microenvironment, composed of cancer cells, surrounding stroma, immune cells, and vascular systems, plays a pivotal role in tumor initiation, progression, metastasis, and the development of treatment resistance (14–16). Emerging research suggests that exercise, through its multisystem physiological effects, can exert profound influence on various regulatory components within the tumor microenvironment, thereby establishing a novel biological foundation for enhancing therapeutic efficacy (17). Exercise has been shown to improve the metabolic state of tumor tissues, increase oxygen delivery efficiency, and alleviate localized hypoxia (18). These changes help to reduce metabolic stress within tumors and inhibit abnormal cellular proliferation. Furthermore, exercise facilitates the redistribution of energy metabolism and alters key metabolic pathways in both tumor cells and their surrounding microenvironment, disrupting the growth advantage of malignant cells (19). From an immunological perspective, exercise enhances immune cell activity and improves their capacity to recognize and eliminate tumor cells (20). It also augments anti-tumor immune responses by optimizing circulatory dynamics, thereby reducing the risk of immune evasion by tumors (21). These regulatory effects collectively contribute to the remodeling of the tumor microenvironment, positioning exercise not merely as an adjunct to therapy but as a critical component in the advancement of personalized cancer treatment.

Given the multidimensional benefits of exercise interventions in cancer care and the growing demand for individualized treatment approaches driven by precision medicine, this review aims to systematically explore the mechanisms, clinical outcomes, and integration potential of exercise interventions in pediatric oncology. With the continued advancement of precision medicine, personalized therapy has emerged as a new direction in cancer treatment (22). Exercise, as a low-risk and sustainable adjunctive modality, is gaining increasing recognition for its therapeutic potential. Through a comprehensive analysis of the physiological, psychological, and immunological effects of exercise in pediatric cancer patients, this review seeks to highlight the unique value of exercise in oncology, particularly its critical role in enhancing treatment efficacy and improving quality of life. Furthermore, this review discusses how exercise interventions may be integrated with precision medicine approaches to provide a theoretical foundation for clinical practice and offer new perspectives for future research. Exercise not only mitigates the adverse effects of cancer therapies but also modulates the tumor microenvironment and immune responses to optimize therapeutic outcomes. As such, exercise is transitioning from a traditional supportive therapy to an essential component of contemporary cancer treatment, with the potential to become a key strategy in personalized oncology. The significance of this review lies in its contribution to the scientific understanding of exercise-based interventions in pediatric cancer care and its support for the clinical implementation of such approaches to help more children overcome the challenges of cancer treatment and achieve better health-related quality of life.

## 2 The unique biological effects of exercise on tumors

### 2.1 Mechanisms of exercise-mediated modulation of the tumor microenvironment

**Figure 1:**
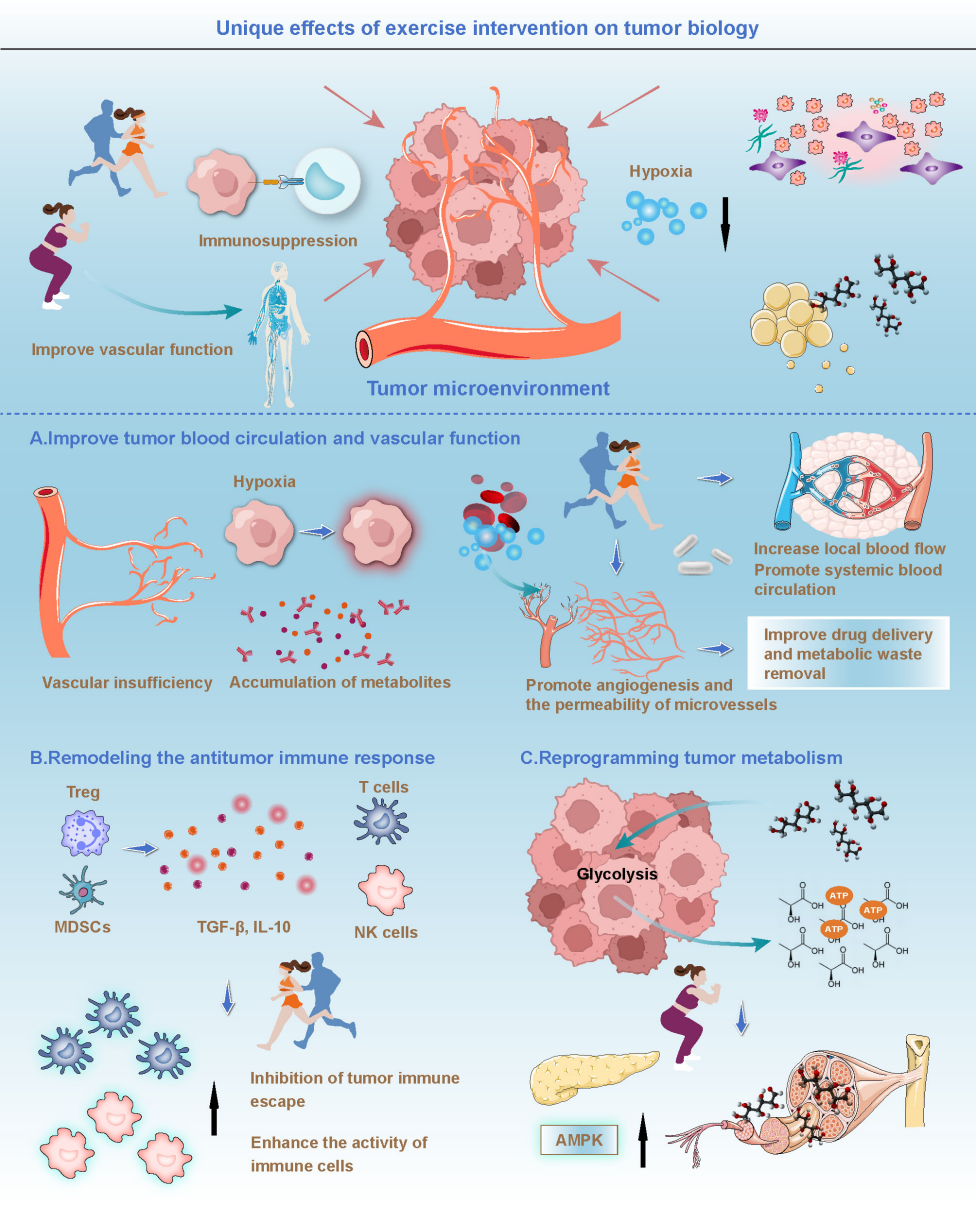
Unique effects of exercise intervention on tumor biology. Upper part: Exercise intervention improves blood circulation and vascular function in the tumor microenvironment, alleviates hypoxia within tumor tissues, promotes blood flow, enhances drug delivery, and facilitates the removal of metabolic waste, thereby improving the effectiveness of tumor treatment. Lower part: **(A)** Improving tumor blood circulation and vascular function: Exercise intervention increases local blood flow, promotes systemic circulation, enhances microvascular permeability, supports oxygenation in tumor regions, and promotes angiogenesis, improving drug delivery and the removal of metabolic waste products. **(B)** Remodeling the antitumor immune response: Exercise enhances the activity of immune cells, reduces the presence of immunosuppressive cells such as regulatory T cells and myeloid-derived suppressor cells, inhibits tumor immune escape, and enhances tumor-specific immune responses. **(C)** Reprogramming tumor metabolism: Exercise enhances AMPK activity, inhibits glycolysis in tumor cells, reduces the tumor cells’ reliance on glucose, improves tumor cell metabolism, and further strengthens the antitumor effects.

The tumor microenvironment is a complex system composed of tumor cells and surrounding vascular, immune, and stromal cells (23). Recent studies have demonstrated that tumor initiation and progression are not solely driven by genetic mutations within tumor cells but are also closely influenced by changes in the tumor microenvironment (24, 25). Interactions among immune cells, blood vessels, and other cellular components within this microenvironment collectively contribute to tumor growth, invasion, and metastasis (26). Exercise, as an intervention capable of influencing systemic physiological states, has been shown to modulate the tumor microenvironment in multiple ways, thereby suppressing tumor growth and metastatic potential (17). One key mechanism involves improving blood circulation within tumor regions (27). Tumor cells depend on rapidly proliferating vasculature to supply oxygen and nutrients for their growth. However, these newly formed vessels are often structurally and functionally abnormal, leading to hypoxia and the accumulation of metabolic byproducts within the tumor (28). Exercise promotes systemic circulation and increases local blood flow, which helps enhance oxygenation in tumor tissues, alleviates hypoxic conditions, and reduces the aggressive proliferation of tumor cells under low-oxygen stress. In addition, exercise supports angiogenesis and improves microvascular permeability, thereby enhancing the delivery of therapeutic agents and facilitating the removal of metabolic waste products from tumor regions (**Figure 1**) (27, 29). Muscle activity during exercise further accelerates circulation, rapidly improving perfusion to tumor sites. This not only increases the supply of nutrients to the tumor area but also enhances the efficiency of drug delivery, ensuring that therapeutic agents can reach tumor cells more directly (30). Evidence suggests that exercise can promote the proliferation of vascular endothelial cells within tumors, improve microvascular structure, and enhance the quality of local blood flow, creating a more favorable physiological environment for subsequent treatment (31).

However, it is crucial to acknowledge that the concept of “vascular normalization” primarily derives from adult epithelial tumor models. Pediatric solid tumors, particularly sarcomas, exhibit distinct vascular architectures. Recent research on tissue-engineered structures highlights the importance of nanotopographic cues in mechanoimmunomodulation, suggesting that the chaotic matrix in sarcomas may respond differently to physical forces (122). Unlike carcinomas, the neovasculature in these tumors often lacks hierarchical organization, necessitating a panvascular perspective to understand how intervention impacts the full-watershed organs (123). Furthermore, given the high prevalence of leukemia in the pediatric population, the hemodynamic effects of exercise extend beyond solid tumor vasculature to the bone marrow niche. Niche environment aberrations are known to promote cancer progression, as seen in other malignancies where the microenvironment shelters tumor cells (124). Exercise intervention has the potential to modulate bone marrow perfusion, thereby disrupting this protective niche. By altering stromal interactions and reducing mitochondrial dysfunction associated with hypoxic stress (125), exercise may force leukemic stem cells out of their dormancy, rendering them more susceptible to systemic treatments. Exercise also plays a significant role in enhancing the immune functionality of the tumor microenvironment. Tumor immune evasion is a key mechanism underlying tumor initiation and progression (32, 33). Tumor cells achieve this by secreting immunosuppressive factors, such as TGF-β and IL-10, which inhibit the immune system’s ability to recognize and eliminate malignant cells (34). Exercise has been shown to regulate immune responses by activating local immune activity and increasing the recruitment and functional capacity of immune cells within tumor regions (20). Research indicates that exercise can elevate both the number and activity of antitumor immune cells, including NK cells, T lymphocytes, and dendritic cells, thereby significantly enhancing antitumor immune responses (**Figure 1**) (35). Furthermore, exercise reduces the presence of immunosuppressive molecules and cell populations within the tumor microenvironment, such as regulatory T cells and myeloid-derived suppressor cells, which are known to impair the function of effector T cells and inhibit antitumor immunity (36). By decreasing the abundance of these immunosuppressive cells, exercise strengthens the immune system’s capacity to target and eliminate tumor cells, thereby effectively suppressing tumor growth. Overall, by improving tumor blood perfusion, enhancing immune cell activity, and normalizing vascular structure within the tumor microenvironment, exercise creates a more favorable biological context for cancer therapy (37).

### 2.2 The relationship between exercise and tumor immune evasion

Immune evasion is a critical mechanism by which tumor cells escape immune surveillance and sustain their growth within the host (38). Tumor cells achieve this by secreting immunosuppressive factors, altering the tumor microenvironment, and inducing immune tolerance, thereby reducing the effectiveness of immune recognition and elimination (39). Exercise, as an effective immunomodulatory strategy, has been shown to counteract tumor immune evasion through multiple mechanisms. By enhancing the activity of immune cells, exercise helps restore immune surveillance against tumor cells (40). Specifically, exercise increases both the number and cytotoxic function of NK cells, which are capable of directly identifying and destroying tumor cells (41). Through the upregulation of NK cell activity, exercise facilitates more efficient elimination of malignant cells and reduces the likelihood of immune escape (42). In addition, exercise promotes the activation and proliferation of T cells, strengthening their antitumor immune functions (43). It also suppresses tumor-mediated immunosuppression through the PD-1/PD-L1 pathway, thereby restoring T cell responsiveness and improving overall antitumor immunity (44). These effects position exercise as a valuable intervention in overcoming immune escape and enhancing immune-mediated tumor control.

Dendritic cells, as key antigen-presenting cells, play a central role in initiating antitumor immune responses by engulfing tumor cells or their secreted antigens and activating tumor-specific T cell responses (45, 46). Studies have shown that exercise can enhance the function of dendritic cells, thereby improving their capacity to present tumor-associated antigens (47). By boosting dendritic cell functionality, exercise contributes to the amplification of tumor-specific immune responses and reduces the extent of immune evasion by tumors (48). In addition, exercise modulates immunosuppressive cells within the tumor microenvironment, further suppressing mechanisms of immune escape (21). The tumor microenvironment is often characterized by a high presence of immunosuppressive cells, including regulatory T cells and myeloid-derived suppressor cells. These cells inhibit effector T cell function through the secretion of immunosuppressive cytokines, thereby impairing the immune system’s ability to recognize and eliminate tumor cells (49). Exercise has been shown to reduce the abundance of these immunosuppressive populations and improve the overall immune activity within the tumor milieu (36). Moreover, it can downregulate the expression of key immunosuppressive molecules such as TGF-β and IL-10, further mitigating immune suppression (50). Overall, exercise enhances the immune system’s responsiveness to tumors through multiple regulatory pathways, suppresses immune evasion, and ultimately contributes to improved antitumor therapeutic efficacy.

### 2.3 Effects of exercise on metabolic pathways and tumor growth

Tumor cells exhibit distinct metabolic characteristics compared to normal cells, with the most notable phenotype being the Warburg effect, in which they preferentially rely on glycolysis for energy production even in the presence of sufficient oxygen (51). Beyond aberrant glucose metabolism, tumor cells also heavily depend on lipid metabolic pathways, including fatty acid synthesis and oxidation, to meet the energy demands and biosynthetic requirements of rapid proliferation (52). As a result, targeting metabolic vulnerabilities in tumor cells and disrupting their energy supply has emerged as a promising anticancer strategy in recent years. Existing studies indicate that exercise can profoundly influence the energy metabolism of tumor cells by modulating systemic metabolic states, thereby inhibiting tumor cell proliferation and growth (19). Exercise enhances skeletal muscle activity and increases glucose uptake efficiency, allowing peripheral tissues to gain greater metabolic access to glucose, which in turn reduces glucose availability for tumor cells and weakens their reliance on glycolysis (**Figure 1**) (53). In addition, exercise improves insulin sensitivity and regulates the insulin and insulin-like growth factor signaling pathways, further limiting the capacity of tumor cells to use glucose for proliferative purposes (54, 55).

Exercise also promotes fatty acid oxidation, thereby reducing excessive accumulation of fatty acids in the body and decreasing the ability of tumor cells to acquire energy and structural substrates through lipogenesis, indirectly inhibiting tumor progression (56, 57). Moreover, the metabolic regulatory effects of exercise extend to other critical pathways by alleviating hypoxia commonly present in the tumor microenvironment, improving tissue oxygenation, and relieving the metabolic stress experienced by tumor cells (58). Exercise also AMPK, a cellular energy sensor that, under energy-deficient conditions, initiates adaptive metabolic responses to suppress non-essential energy consumption and biosynthesis, thereby reducing the metabolic burden on cells (59). By enhancing AMPK activity, exercise not only restricts the energy supply available to tumor cells but also diminishes their proliferative capacity, reinforcing the antitumor effects of physical activity (60).

Nevertheless, a critical physiological distinction between pediatric and adult oncology is the patient’s metabolic state; children are in an active phase of somatic growth requiring an anabolic metabolism. Similar to the complex requirements seen in tissue healing and tendon-bone repair, pediatric physiology demands a robust energy supply for structural regeneration (126). While exercise-induced AMPK activation effectively inhibits tumor glycolysis, it may antagonize pathways essential for normal development. This presents a unique therapeutic challenge: exercise prescriptions must be calibrated to induce metabolic stress on the tumor without disrupting the delicate metabolic balance required for growth, a concept supported by population studies linking genetic variants to metabolic control (127).

## 3 Exercise interventions and their impact on physiological and psychological health in pediatric cancer patients

### 3.1 The role of exercise in physiological recovery

Structured exercise interventions can play a vital role in supporting the recovery of physiological functions in pediatric cancer patients and serve as an important approach to improving overall rehabilitation outcomes. By enhancing muscular strength and function, exercise significantly contributes to mitigating progressive muscle atrophy during treatment (61). Prolonged bed rest or reduced physical activity often results in substantial declines in muscle mass and endurance (62). In contrast, regular physical activity stimulates muscle protein synthesis and fiber regeneration, thereby promoting strength restoration and improving physical capacity (63). Exercise interventions also provide mechanical loading to the skeletal system, which facilitates mineral deposition and increases bone mineral density (64). These effects help reduce the risk of osteoporosis induced by chemotherapy and radiotherapy while improving the structural stability of the bones. Such benefits are particularly important for children undergoing critical periods of growth and development.

The cardioprotective effects of exercise also warrant attention. Anticancer treatments are often associated with declines in cardiac function and reduced vascular elasticity (65). Aerobic exercise can improve pulmonary ventilation and circulatory efficiency, enhance myocardial tolerance, and elevate overall cardiopulmonary performance (66). By promoting blood circulation and oxygen delivery, exercise helps to reduce the additional burden placed on the cardiovascular system during treatment and lowers the risk of long-term cardiac complications (67). In addition, exercise contributes positively to weight management and helps prevent metabolic abnormalities resulting from physical inactivity, including obesity, insulin resistance, and dyslipidemia (68). Overall, by improving the function of the muscular, skeletal, and cardiovascular systems, exercise interventions can effectively alleviate treatment-related adverse effects and enhance the overall physiological health of pediatric cancer patients.

### 3.2 The impact of exercise on psychological health

Pediatric cancer patients commonly experience varying degrees of psychological distress during treatment, including anxiety, depression, fear, and feelings of isolation (69). Persistent psychological burden not only affects treatment adherence but may also hinder overall recovery and reduce quality of life (70). Exercise interventions have been shown to improve mental health through multiple mechanisms and represent an important behavioral strategy for promoting emotional stability. Physical activity can significantly alleviate symptoms of anxiety and depression in children undergoing cancer treatment (71). Factors such as treatment uncertainty, physical discomfort, and concerns about the future often contribute to heightened psychological stress (72). Exercise helps regulate emotional responses by stimulating the release of neurotransmitters such as endorphins, which act on mood-regulating centers in the brain (73). Elevated endorphin levels are associated with reduced anxiety and enhanced feelings of well-being, thereby increasing patients’ psychological resilience and acceptance of the treatment process.

Exercise plays a crucial role in enhancing self-efficacy. Under the dual pressures of prolonged treatment and physical discomfort, pediatric patients often experience feelings of helplessness and loss of control (74). Regular physical activity offers structured, attainable goals that allow patients to regain a sense of competence and personal agency through the progressive accomplishment of exercise tasks (75). Improvements in physical capacity and emotional well-being that accompany consistent exercise can further alleviate psychological burden and enhance patients’ resilience and coping abilities in the face of illness (76). Therefore, exercise is not only a means of restoring physical function but also a valuable approach to improving mental health and fostering a more positive attitude toward treatment.

### 3.3 Supportive effects of exercise on cognitive function

Cognitive impairment is a common side effect observed in pediatric cancer patients undergoing chemotherapy and radiotherapy, often referred to as chemotherapy-related cognitive dysfunction. These impairments may manifest as reduced attention span, memory decline, and slower information processing speed, significantly affecting children’s academic performance and daily functioning (77). With growing recognition of the neurocognitive benefits of exercise, increasing evidence suggests that physical activity can partially reverse or mitigate these cognitive deficits. Regular exercise, particularly aerobic activity, has been shown to improve cognitive function by enhancing neuroplasticity (78). Exercise increases cerebral blood flow, thereby improving the delivery of oxygen and nutrients to the brain, which supports neuronal survival, differentiation, and functional maintenance (79). Moreover, exercise significantly upregulates the expression of BDNF, a critical molecule involved in neuronal growth, synaptic plasticity, and cognitive processes such as learning and memory (80). Elevated BDNF levels promote synaptic connectivity and facilitate cognitive recovery, especially in domains related to attention and memory (81). In addition to these direct neurological effects, exercise also contributes to cognitive improvement by indirectly enhancing emotional well-being and sleep quality (82). Mental health is closely linked to cognitive performance, and negative emotional states such as anxiety and depression can exacerbate cognitive dysfunction (83). By alleviating psychological stress and improving sleep, exercise fosters an environment conducive to neural recovery, thereby enhancing concentration and learning efficiency. Sleep improvement is particularly important, as it plays a vital role in memory consolidation and cognitive processing (84). In summary, exercise supports cognitive function recovery in pediatric cancer patients through both direct mechanisms involving neuroplasticity and indirect pathways related to mood and sleep, ultimately contributing to improved learning capacity and quality of life.

## 4 Integration of exercise intervention with personalized treatment

### 4.1 Designing exercise interventions based on patient-specific differences

Against the backdrop of advancing precision medicine, treatment strategies for pediatric cancer patients must account for multidimensional differences, including pathological features, gene expression profiles, and immune status (85). Significant heterogeneity exists among patients in terms of tumor biology, oncogenic driver mutations, and immune responsiveness, rendering traditional standardized treatment approaches insufficient to meet individualized therapeutic needs (86). Designing exercise interventions based on each patient’s unique pathobiological characteristics not only supports physiological recovery but also has the potential to enhance treatment responsiveness, mitigate adverse effects, and improve overall therapeutic outcomes.

**Figure 2:**
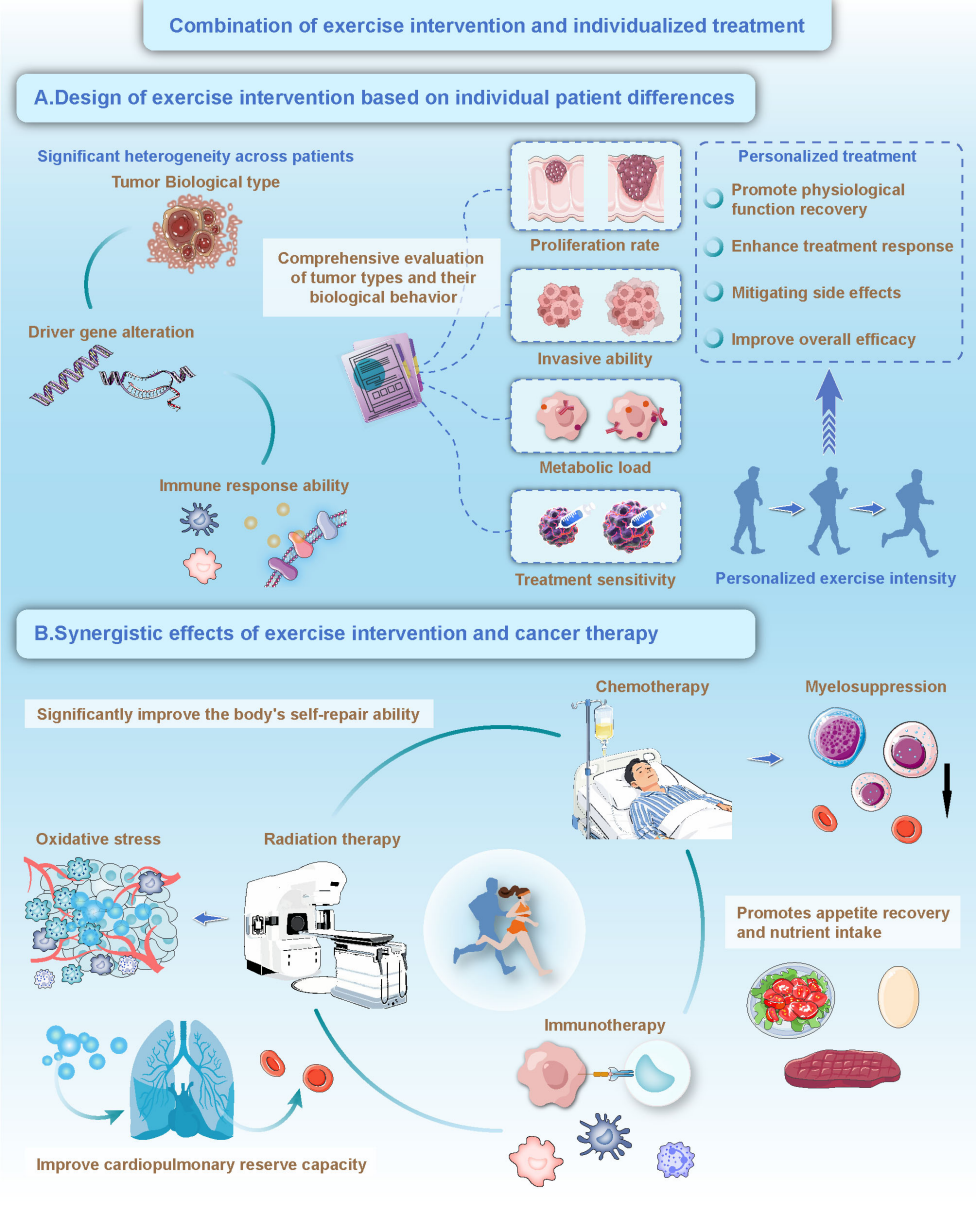
Combination of exercise intervention and individualized treatment. **(A)** Design of exercise intervention based on individual patient differences: Exercise interventions should be designed based on individual patient differences, considering factors such as tumor biological type, proliferation rate, invasive ability, metabolic load, immune response ability, treatment sensitivity, and driver gene alteration, with personalized exercise intensity to promote physiological function recovery, enhance treatment response, mitigate side effects, and improve overall efficacy. **(B)** Synergistic effects of exercise intervention and cancer therapy: Exercise intervention significantly improves the body’s self-repair ability, enhances the effects of chemotherapy, radiation therapy, and immunotherapy, reduces oxidative stress, improves cardiopulmonary reserve capacity, alleviates myelosuppression, and promotes appetite recovery and nutrient intake, thereby synergistically enhancing cancer treatment outcomes.

When designing personalized exercise interventions, it is essential to comprehensively assess the tumor type and its biological behavior (**Figure 2**). Different cancers vary significantly in terms of proliferation rate, invasiveness, metabolic burden, and treatment sensitivity, necessitating tailored approaches to exercise type, intensity, and frequency. For instance, patients with leukemia often experience profound fatigue and muscle weakness due to bone marrow suppression and may benefit from low-intensity exercise aimed at maintaining basic functional capacity (87). In contrast, patients with solid tumors may be better suited to moderate-intensity exercise programs designed to improve muscular strength and cardiopulmonary function (76).

Furthermore, for patients with primary bone tumors such as osteosarcoma, safety concerns regarding mechanical loading must be addressed. While exercise benefits represent the consensus, the theoretical risk that excessive physical stress or increased interstitial fluid pressure might promote cell dislodgement or micrometastasis warrants caution, necessitating strictly controlled loading protocols.

Advances in genomics have provided a new theoretical foundation for personalized exercise interventions (88). Specific genetic mutations may influence factors such as muscle repair capacity, exercise tolerance, or metabolic adaptability, thereby affecting the optimal intensity and modality of physical activity (89, 90). For example, children with mutations affecting mitochondrial function may require gentler forms of exercise to prevent excessive oxidative stress (91, 92). Moreover, high-risk driver mutations offer specific targets for intervention. Research into germline pathogenic variants demonstrates how specific genetic alterations can dictate disease phenotype and progression risk (128). Similarly, alternative RNA splicing events in cardiac and systemic diseases highlight the molecular complexity that exercise might influence (129). For instance, MYCN-amplified tumors exhibit distinct metabolic plasticity. This specific dependency renders them potentially more sensitive to exercise regimes that target these metabolic vulnerabilities. By aligning exercise intensity to target such genotype-specific characteristics, clinicians can move from generic rehabilitation to precise, biologically-driven therapy. In addition, the patient’s immune status is a critical factor in designing individualized exercise protocols. While exercise can enhance immune cell function, patients with severely compromised immunity may benefit more from gradual, low-intensity activity, whereas those with relatively stable immune function may tolerate higher intensities that promote immune activation (93).

### 4.2 Synergistic effects of exercise intervention with cancer therapies

Exercise interventions not only independently improve the physiological and psychological well-being of pediatric cancer patients but also interact synergistically with conventional treatments such as chemotherapy, radiotherapy, and immunotherapy, thereby significantly enhancing overall therapeutic efficacy (94). Although traditional cancer therapies are effective in eliminating malignant cells, they are frequently accompanied by severe side effects including immunosuppression, bone marrow suppression, fatigue, and malnutrition, which can hinder recovery and diminish quality of life (95). Exercise, as a safe and sustainable intervention, can enhance treatment outcomes and reduce adverse effects through multiple biological pathways (**Figure 2**). During chemotherapy and radiotherapy, exercise supports the body’s capacity for self-repair (96). Chemotherapy-induced bone marrow suppression often results in reduced white blood cell counts and weakened immune function, while regular physical activity promotes hematopoietic system recovery by increasing the production of white and red blood cells, thereby alleviating immunosuppression (97). Despite these synergistic benefits, the timing of exercise intervention must be strictly coordinated with chemotherapy cycles. Pediatric protocols often induce profound lymphodepletion, leading to a “nadir” phase where the patient’s immune defense is critically compromised. According to the “open window” theory, high-intensity exercise during this period may transiently decrease immune cell concentration and increase infection risk. Therefore, a phase-dependent exercise prescription is necessary: maintaining low-intensity activity during the nadir phase to preserve function, while reserving higher-intensity protocols for the recovery phase. Radiotherapy generates reactive oxygen species that damage healthy tissue (98). Exercise strengthens the endogenous antioxidant defense system, thereby reducing oxidative stress and minimizing radiation-related injury (99). Improved cardiopulmonary reserve through exercise helps relieve excessive fatigue caused by treatment, enhances therapy tolerance, and supports physical performance (100). Furthermore, exercise contributes to appetite restoration and improved nutritional intake, offering essential metabolic support to aid patient recovery during intensive treatment (101).

With the growing application of immunotherapy, the synergistic effects between exercise and immune-based treatments have become an emerging focus of research. Immunotherapy relies on the patient’s own immune response to eliminate tumor cells, but its efficacy is often limited by the immunosuppressive characteristics of the tumor microenvironment (102). Exercise can enhance immune activity by activating natural killer cells and T cells, while simultaneously reducing the accumulation of immunosuppressive cells such as regulatory T cells, thereby improving the overall responsiveness to immunotherapy. In addition, exercise may help alleviate immunotherapy-related adverse effects, increase treatment tolerance, and lower the risk of premature treatment discontinuation (12, 103). The complementary relationship between exercise and therapies such as chemotherapy, radiotherapy, and immunotherapy not only enhances the comprehensive efficacy of cancer treatment but also supports the physiological and psychological recovery of patients, making the treatment process more manageable and sustainable. As research into the biological mechanisms of exercise continues to expand, its potential application in personalized therapy is expected to grow, positioning exercise as a routine component of integrated pediatric cancer care in the future.

## 5 Clinical research and implementation challenges of exercise intervention

### 5.1 Clinical evidence and outcome evaluation of exercise intervention

Exercise intervention, as a supportive therapeutic approach, has gained increasing recognition in recent years for its potential value in pediatric oncology. Numerous clinical studies have shown that appropriate exercise programs can effectively improve the physical condition of pediatric patients, alleviate various treatment-related side effects, enhance immune function, and contribute to the overall improvement of quality of life (5). However, due to the significant heterogeneity among pediatric cancer patients in terms of age, developmental stage, psychological status, and treatment burden, the scientific evaluation of exercise outcomes remains challenging. Existing evidence suggests that different forms of exercise interventions have varying effects on patients (104). Aerobic activities such as walking, running, and swimming have demonstrated substantial rehabilitative benefits by enhancing cardiopulmonary endurance, improving blood circulation, and reducing fatigue (105). Clinical trials have indicated that during the recovery phase following chemotherapy and radiotherapy, aerobic exercise can significantly relieve fatigue and improve physical activity levels (106). Resistance training primarily targets the musculoskeletal system, helping to increase muscle strength, improve muscle quality, and reduce skeletal fragility, which is particularly relevant in addressing chemotherapy-induced muscle atrophy and osteoporosis (107). Further research has found that combining aerobic and resistance training can produce more comprehensive synergistic effects, including improvements in physical fitness, weight stability, cardiopulmonary function, and skeletal health (108). Commonly used clinical tools for evaluating exercise effectiveness include physical fitness tests, quality of life questionnaires, and physiological monitoring such as heart rate, blood pressure, and lung capacity (109). To advance the scientific rigor of exercise oncology, future trials must move beyond these standard clinical outcomes. We propose the integration of deep phenotyping protocols, similar to those used in the Human Phenome Project, to extract high-dimensional imaging and physiological phenotypes (130). Furthermore, evaluating the mediating role of inflammation in health outcomes is crucial (131). We recommend monitoring mechanistic biomarkers, such as hemodynamic stability indicators (132) and specific inflammatory mediators, to quantify the biological efficacy of exercise interventions comprehensively. Nonetheless, current evaluation frameworks remain limited, particularly in quantifying treatment benefits for pediatric patients and in developing personalized assessment models. As clinical evidence continues to grow, there is an urgent need to establish standardized evaluation systems specifically tailored to children with cancer to enhance the comparability and scientific rigor of exercise intervention research.

### 5.2 Implementation barriers and potential solutions for exercise intervention

Although the clinical potential of exercise interventions has been increasingly recognized, their implementation in real-world settings continues to face multiple challenges. One of the primary barriers is patient participation. Pediatric cancer patients often experience symptoms such as nausea, vomiting, pain, and fatigue during treatment, which may lead to reluctance or resistance toward physical activity (110). This issue is particularly pronounced among younger children who lack the ability to self-regulate, making it difficult to maintain motivation and engagement in exercise programs (111). Enhancing interest and compliance in this population remains a significant challenge for effective intervention delivery. Another limiting factor is the lack of resources. Many healthcare institutions do not have access to specialized exercise therapy facilities or adequately trained exercise professionals. Given the extended duration of cancer treatment and the rapidly changing physical condition of pediatric patients, designing and adapting exercise programs requires strong clinical expertise and interdisciplinary collaboration. In settings with limited resources, developing scientifically sound, feasible, and cost-effective exercise strategies poses a critical challenge for clinical practice.

Addressing these barriers requires a comprehensive, multi-level approach. Enhancing education for patients and their families can improve understanding of the benefits of exercise interventions and increase willingness to participate (112). Designing exercise programs that are both engaging and feasible for children of different age groups can help reduce resistance and promote sustained involvement (113). Healthcare institutions should prioritize the training of professionals such as exercise therapists and physical therapists, and establish interdisciplinary teams to provide safe, precise, and personalized exercise guidance for pediatric cancer patients (114). Additionally, leveraging telemedicine platforms to deliver home-based guidance, virtual consultations, and remote exercise monitoring can effectively extend the reach of interventions in resource-limited settings (115). Future research should also focus on the dynamic physiological and psychological changes that occur during the course of treatment in pediatric patients. Longitudinal monitoring of these changes can provide valuable insights for the development of individualized exercise plans. Establishing practical exercise prescription frameworks and clinical pathways will be essential for integrating exercise interventions into routine pediatric oncology care.

## 6 Conclusion and future directions

### 6.1 A systematic summary: redefining exercise as an integral component of pediatric cancer care

Exercise intervention is increasingly evolving from a traditional rehabilitative measure to an integral component in the design of comprehensive treatment strategies for pediatric oncology. This review systematically examines the role of exercise in pediatric cancer care across multiple dimensions, including tumor biology, microenvironmental regulation, metabolic reprogramming, immune modulation, and the restoration of physical and psychological function. Current evidence indicates that exercise interventions can alleviate physiological impairments commonly associated with chemotherapy and radiotherapy, such as muscle atrophy, bone loss, reduced cardiopulmonary capacity, and persistent fatigue. At the same time, exercise has demonstrated significant benefits in mitigating psychological challenges such as anxiety, depression, fear, and helplessness, while also contributing to the recovery of cognitive functions, including attention, memory, and information processing. More importantly, exercise enhances blood perfusion and oxygenation in tumor regions, promotes the exchange of nutrients and metabolic byproducts, and alleviates local hypoxia and acidosis, thereby contributing to the macro-level optimization of the tumor microenvironment. Furthermore, exercise influences glucose metabolism, fatty acid oxidation, and energy-sensing pathways, reducing the preferential access of tumor cells to energy substrates and biosynthetic precursors within the systemic metabolic network, which in turn diminishes their proliferative advantage. Collectively, this multidimensional evidence supports the view that exercise interventions in pediatric cancer care are grounded in clear biological mechanisms rather than being merely empirical rehabilitation tools.

Building on this foundation, the present review proposes an overarching conceptual framework in which exercise intervention may reshape the treatment paradigm for pediatric oncology. On one hand, exercise enhances antitumor immune responses by promoting the activation of natural killer cells, effector T cells, and dendritic cells, while suppressing the abnormal accumulation of regulatory T cells and myeloid-derived suppressor cells, thereby alleviating immunosuppression and strengthening immune-mediated tumor control. On the other hand, exercise improves vascular structure and function, creating more favorable pathways for the delivery of chemotherapeutic agents, radiotherapeutic energy, and immunotherapeutic molecules, thereby amplifying the efficacy of conventional treatments (116). At the clinical level, exercise interventions improve cardiopulmonary reserve, physical capacity, and nutritional status, which collectively enhance pediatric patients’ tolerance to intensive treatment regimens and reduce the risk of treatment interruption or dose reduction due to adverse effects. This, in turn, increases treatment completion rates and overall therapeutic benefit. The review therefore emphasizes that exercise should be regarded as a systemic intervention strategy that spans the entire course of pediatric cancer treatment. Its value lies not only in alleviating isolated adverse effects but also in integrating microenvironmental remodeling, metabolic regulation, and immune activation to support a more holistic and forward-looking model of comprehensive cancer care.

### 6.2 Future research priorities and innovative directions

Looking toward the future, the continued advancement of exercise interventions in pediatric oncology requires the deep integration of basic science and clinical research. First, at the mechanistic level, numerous critical scientific questions remain unresolved. For example, it is not yet clear whether exercises of varying intensities and modalities produce distinct effects on the tumor microenvironment, remodel immune cell subset composition, or modulate tumor metabolic vulnerabilities. The optimal timing for implementing exercise interventions across different phases of tumor initiation, progression, and recurrence also remains to be clarified. Furthermore, it is essential to determine whether children with different tumor types and oncogenic driver gene profiles exhibit differential biological responses to exercise. Addressing these issues will support a shift from empirical, uniform exercise protocols to mechanism-informed, stratified intervention paradigms. Second, the use of multi-omics approaches, including transcriptomics, proteomics, metabolomics, and immunomics, is essential for identifying biomarkers that can predict exercise sensitivity and therapeutic benefit (117). These biomarkers will inform the development of models that guide both the design and dynamic adjustment of exercise prescriptions. Such high-dimensional strategies may facilitate a gradual transition from standardized exercise recommendations to truly personalized exercise regimens.

At the clinical and practical levels, the future development of exercise interventions in pediatric oncology calls for multi-dimensional collaborative efforts. On one hand, there is a need to conduct more prospective, randomized controlled trials with long-term follow-up specifically focused on pediatric cancer populations. These studies should systematically assess the effects of exercise interventions on key outcomes such as survival, recurrence, long-term cardiovascular and metabolic complications, psychological recovery, and the reintegration of educational and social functioning. Such research is vital to address current gaps related to limited sample sizes, insufficient follow-up durations, and suboptimal endpoint selection. On the other hand, it is essential to explore the integration of exercise with chemotherapy, radiotherapy, immunotherapy, and targeted therapies within real-world clinical settings. This integration should aim to develop combined intervention protocols tailored to specific cancer types and treatment phases. The use of wearable devices, remote monitoring platforms, and intelligent exercise management systems may enable continuous observation and adaptive modulation of exercise behaviors, especially in contexts where healthcare resources are limited or geographic disparities exist. These technologies can enhance both the feasibility and adherence of exercise prescriptions (118–121). Moreover, establishing interdisciplinary teams composed of pediatric oncologists, rehabilitation specialists, exercise physiologists, nutritionists, and psychologists, supported by coherent health policies and reimbursement frameworks, will facilitate the transition of exercise interventions from pilot applications to routine clinical practice. Through collaborative progress along these interconnected pathways, exercise is positioned to play a strategic role in comprehensive pediatric oncology care by supporting the dual objectives of improved survival and enhanced quality of life.
